# Annotation

**Published:** 1956-04

**Authors:** 


### Annotation

### BRISTOL REGISTER OF CONGENITAL HEART DISEASE

It is only comparatively recently that the exact diagnosis of congenital heart disease has become more than an academic exercise and a guide to prognosis.

Since Gross tied the first patent ductus in 1939 the achievements of surgery have been spectacular and it has become possible to treat many of these conditions. The advent of the heart-lung machine will further extend the scope of surgery. In some cases a complete cure is possible or feasible. The ligature of a patent ductus, the closure of a defect in the auricular or ventricular septa or the division of a stenotic pulmonary valve leave an essentially normal heart. In others the defect is too gross to be corrected surgically but some readjustment of the circulation can be made that produces some improvement, tricuspid atresia, Fallot's tetralogy and perhaps transposition of the great vessels come into this category. In another group of gross anomalies, truncus arteriosus, neither surgical correction or palliation seem possible.

If surgery is to be contemplated in these conditions an exact diagnosis must be made, and in many cases this cannot be done without the aid of the special techniques of cardiac catheterization and angiocardiography.

Not only must an exact diagnosis be made, but an exact appreciation of the natural course of the disease is necessary in order to know whether the risks of surgery outweigh the risks of doing nothing. For example, it is now possible to close ventricular septal defects using a heart-lung machine, but many, perhaps the majority, of these patients live a normal span without disability. But some patients run into trouble and need treatment, and it is often difficult to decide exactly when surgical treatment should be advised. Again, some operations will result not in a normal heart, but in a new lease of life which may be long or short, how long or how short remains to be seen.

It is obviously necessary to keep careful records of all cases of congenital heart disease whether treated or untreated in order to know what we should do, when we should do it and what we should leave undone.

For these reasons it has been decided to keep a register of all cases of congenital heart disease seen at the United Bristol Hospitals. Over the course of years a valuable fund of knowledge will be accumulated.

Cushing, a great keeper of statistics, once said to an Englishman who had come to work with him: " There wouldn't be much point in playing cricket if you didn't keep the score ". We intend not only to keep the score, but the batting and bowling averages as well.

